# Implementing patient blood management in a Brazilian public women’s hospital: a pre–post implementation study

**DOI:** 10.1016/j.bjane.2026.844839

**Published:** 2026-10-01

**Authors:** Lorena Costa Corrêa, Nataly Andrade Alves de Paiva, Ana Carolina Dias Almeida, Ulisses Montarroyos, Cesar de Almeida-Neto

**Affiliations:** a Universidade de Pernambuco, Centro Universitário Integrado Amaury de Medeiros, Recife, PE, Brazil; b Universidade Federal de Pernambuco, Hospital das Clínicas Romero Marques, Recife, PE, Brazil; c Universidade de Pernambuco, Programa de Pós-Graduação da Faculdade de Ciências Médicas, Recife, PE, Brazil; d Faculdade de Medicina da Universidade de São Paulo, Disciplina de Pós-Graduação em Ciências Médicas, São Paulo, SP, Brazil; e Fundação Pró-Sangue, Hemocentro de São Paulo, São Paulo, SP, Brazil

**Keywords:** Anemia, iron-deficiency, Blood transfusion, Bloodless medical and surgical procedures, Hysterectomy, Postpartum hemorrhage, Preoperative care

## Abstract

**Background:**

Patient Blood Management (PBM) is a personalized, evidence-based, and cost-effective approach. In surgery, the objective is to detect and treat anemia, minimal perioperative blood loss, and promote restrictive transfusion practices. It improves clinical outcomes, patient engagement, and reduces costs. We evaluated the effect of PBM on preoperative management of hysterectomies for uterine fibroids and Postpartum Hemorrhage (PPH) in a Brazilian public hospital.

**Methods:**

In this pre-post intervention study, a PBM outpatient clinic was established, a Hemorrhage Management Protocol (HMP) was implemented, and low-cost educational initiatives were used. The study period was from 2016 to 2023.

**Results:**

Overall, 36 patients were included; twenty-two returned to a 30-day appointment and had a mean preoperative hemoglobin increase of 2.48 g.dL^-1^ (95% Confidence Interval: 1.8–3.16; p < 0.001). Twenty-five underwent surgery. No preoperative prophylactic transfusions were administered. Transfusions of Packed Red Blood Cells (PRBC) had a mean reduction of -77.4 (p = 0.253) before January 2020 and maintained its behavior until 2023 (1.9; p = 0.686). The HMP was activated in five cases for PPH and 23 units of PRBCs and 11 units of Fresh Frozen Plasma (FFP) were transfused (mean of 4.6 PRBC units and 2.2 FFP units per patient). Additionally, 546 healthcare professionals accessed the PBM guidelines via instant messaging during the study period.

**Conclusion:**

The implementation of PBM by leveraging existing resources led to appropriate treatment of iron deficiency anemia before hysterectomies; systematic use of hemocomponents for PPH and sustained reduction on PRBC transfusions.

## Introduction

Patient Blood Management (PBM) is a personalized, evidence-based, and cost-effective approach with three well-defined pillars: early detection and treatment of anemia and minimal blood loss in the preoperative period with restricted transfusion practices. These strategies have improved clinical outcomes, increased patient engagement, and reduced costs.^1^^,^^2^ This care model emerged in 2005 and was globally recommended in 2010 by the World Health Organization (WHO), which called for the urgent global implementation of PBM by 2021.^1^^,^^3^^,^^4^

PBM may be applied for the management of preoperative anemia in surgeries for uterine myomatosis, Postpartum Hemorrhage (PPH), and postoperative or postpartum scenarios. Preoperative correction of anemia with oral or intravenous iron supplementation is recommended for patients undergoing hysterectomy or myomectomy. Several measures can be adopted to prevent and manage PPH. In the postoperative or postpartum period, for stable patients with anemia, tolerance to anemia is achieved by using antihemorrhagic drugs and micronutrients (e.g., iron) and restricting allogeneic blood transfusions to only required cases.^5^, ^6^, ^7^, ^8^

Fibroids are benign myometrial tumors with an incidence of 5%–65%; surgical treatment is indicated in 80%–90% of cases. Women with fibroids develop Iron-Deficiency Anemia (IDA), which causes loss of quality of life, absence from work, and job losses.^5^^,^^8^ Iron replacement is indicated to normalize Hemoglobin (Hb) levels, correcting anemia and stopping or reducing abnormal menstrual bleeding. There is no clinical indication for the prophylactic use of blood components in the preoperative setting. However, in our country, it is common for hospitalized patients with anemia who are scheduled to undergo myomectomy or hysterectomy to receive Packed Red Blood Cell (PRBC) transfusions with the aim of achieving preoperative hemoglobin levels of 10–12 g.dL^-1^. Nevertheless, this approach is associated with poor clinical outcomes.^6^^,^^9^, ^10^, ^11^, ^12^

PPH is the loss of ≥ 500 mL of blood in the third stage of labor for vaginal delivery or ≥ 1,000 mL for cesarean delivery, occurring up to 48h postpartum.^13^ It is an important cause of maternal death worldwide and the second one in Brazil. In Pernambuco, PPH caused 12% of maternal deaths in 2018.^14^ Therefore, it is essential to establish well-defined, evidence-based, and readily applicable Hemorrhage Management Protocols (HMPs).^13^, ^14^, ^15^ Additionally, the administration of tranexamic acid within three hours of the diagnosis of Postpartum Hemorrhage (PPH), along with the implementation of an HMP, is recommended and may improve patient outcome.^16^^,^^17^

Until 2016, the preoperative routine at our institution included the transfusion of PRBCs before hysterectomy, with the aim of increasing hemoglobin levels prior to surgery. Although Brazilian Postpartum Hemorrhage (PPH) protocols were followed, no standardized Massive Transfusion Protocol (MTP) was in place, and Fresh Frozen Plasma (FFP) was administered routinely without an evidence-based indication. Therefore, to assess the impact of a Patient Blood Management (PBM) program on transfusion practices, we reviewed strategies for managing preoperative anemia in patients with uterine myomatosis, implemented an MTP, and promoted a more restrictive, tolerance-based approach to anemia. In addition, we encouraged restrictive transfusion practices and the use of specific therapeutic algorithms for anemia management. To support the management of preoperative anemia and optimize transfusion practices, we implemented low-cost, rapid, and widely accessible educational measures. These interventions addressed all three pillars of PBM using the resources available at our institution.

## Methods

### Type of study

This was an intervention study with pre- and post- evaluations, including the implementation of PBM. As part of this program, three procedures were introduced concurrently at Centro Universitário Integrado de Saúde Amaury de Medeiros (CISAM) based on the pillars of PBM (Fig. 1).

**Figure 1:**
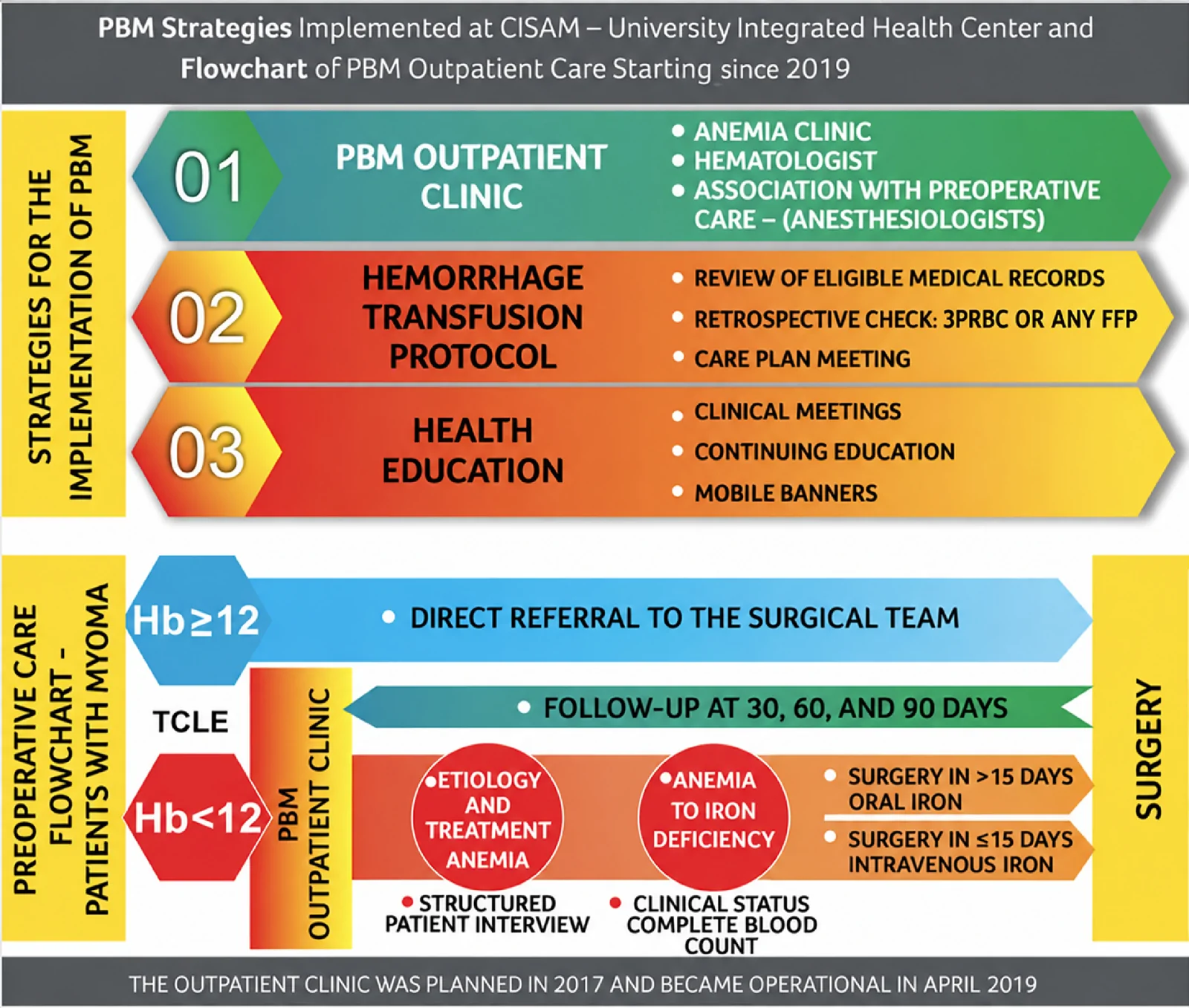
PBM strategies implemented at the Integrated University Health Center (CISAM) and the flow chart of care at the CISAM PBM outpatient clinic as of January 2020.

### Study setting

CISAM, linked to the University of Pernambuco (UPE), is a leading institution for women's health located in the northeastern region of Brazil. It has 108 beds and serves the Unified Health System. This unit is part of the UPE Hospital Complex (CH/UPE), which consists of 839 beds. CISAM provides outpatient care to approximately 80 women scheduled for fibroid correction. It conducts averagely 280 gynecological surgeries monthly, of which 25 are uterine myomatosis corrections. CISAM conducts 350 deliveries per month, with a PPH rate of 3%–5% and 1% requiring blood transfusions. As a teaching center, it receives students and residents from medical, nursing, and multidisciplinary areas.^18^

Our institution uses semi-electronic medical records and has no access to cell salvage systems or point-of-care testing.

### Study period

The study was conducted from December 2016 to December 2023. The PBM program was implemented on 31^st^ December 2019, when an outpatient clinic, a hemorrhage management protocol, and educational measures were implemented concurrently.

#### Procedure 1 ‒ Patient blood management outpatient clinic

We identified and treated anemia for patients scheduled for hysterectomy or myomectomy. Patients with Hemoglobin (Hb) levels < 12 g.dL^-1^ who were scheduled for elective hysterectomy or myomectomy were evaluated and followed at the PBM outpatient clinic of CISAM (Fig. 1). Before the initial consultation, all patients were invited to participate in the study and were given the opportunity to ask questions and withdraw from the study at any time. Written informed consent was obtained from all participants.

At the initial consultation, a structured medical history was obtained, including anemia-related clinical data, menstrual parameters, and information suggestive of other potential causes of anemia. Patients with Abnormal Uterine Bleeding (AUB) or ultrasound-confirmed uterine fibroids, Hb < 12 g.dL^-1^, low Mean Corpuscular Volume (MCV), and/or elevated Red cell Distribution Width (RDW) were considered to have iron-deficiency anemia, even when a complete blood count was the only available laboratory test. This diagnostic approach was adopted because of limited resources and the lack of routine iron studies, in conjunction with a well-documented clinical history and the absence of other conditions associated with bleeding.

Patients diagnosed with anemia were treated according to the interval between the consultation and the scheduled surgery. When surgery was scheduled more than 15 days after the consultation, patients received oral iron therapy prescribed by their attending physicians, at a dose of 100–200 mg of elemental iron per day. When surgery was scheduled within 15 days, intravenous iron therapy with iron sucrose was administered, with the dose calculated according to the Ganzoni formula (Fig. 1).^19^ The number of infusions depended on the total iron requirement to correct iron deficiency and anemia. Patients were followed up on day 30, 60 and 90 from the first appointment. A hemoglobin increase of ≥ 1 g.dL^-1^ within 30 days was considered a satisfactory response to iron replacement therapy. Patients who showed an inadequate response to oral iron or were intolerant to it were subsequently treated with intravenous iron. Patients presenting symptoms or signs suggestive of neoplasia were referred for specialist evaluation. No cases of malignancy or hereditary anemia were diagnosed. To assess perioperative PRBC utilization, we reviewed the medical records of patients undergoing surgery at CISAM from 48 hours prior to surgery until hospital discharge.

Transfusion was defined as each episode of blood component administration. PRBC transfusion was quantified in units, whereas FFP and platelet transfusions were recorded as transfusion episodes. The total number of blood components administered was recorded in units. For bleeding control, all patients received tranexamic acid and hormonal therapy when indicated by the attending gynecologists. No intraoperative interventions were performed during elective procedures.

#### Procedure 2 ‒ Implementation of the hemorrhage management protocol

The Hemorrhage Management Protocol used at hospital for PPH is based on Brazilian's Ministry of Health, which has recommendations on use of oxytocin for prevention and tranexamic acid for treatment after diagnosis of PPH. Despite this guide, the massive transfusion protocol (MTP) was not in place. So, we designed it based on the Australian hemorrhage and transfusion protocols and included it in the clinical guidelines in 2019. At that time, Australian protocol for major hemorrhage control recommended use 1:1:1 ratio of PRBC:FFP: Platelet, or 2:1:1, considered the use of tranexamic acid for PPH and use of cryoprecipitate if bleeding continued, or if fibrinogen < 1 lt; 1 g.L^-1^ (100 mg.dL^-1^).^20^ So, at CISAM, MTP was designed in 2019 to be activated in cases of administration of four or more PRBC transfusions in < 4h by the direct patient care team (Fig. 2). If the Transfusion Agency (TA) team identifies a situation that fits its parameters, the responsibility for conducting the procedure falls on the TA.^21^^,^^22^ To analyze retrospectively MTP activation for PPH, we considered an initial searching of records from patients who have received three or more units of PRBC and at least one unit of FFP infusion (Supplementary Materials Fig. 1). After that, all patients who have received four or more PRBC transfusions (met MPT activation criteria) and had the diagnosis of PPH were included for this study (Supplementary Materials Figs. 1 and 2).^13^

**Figure 2:**
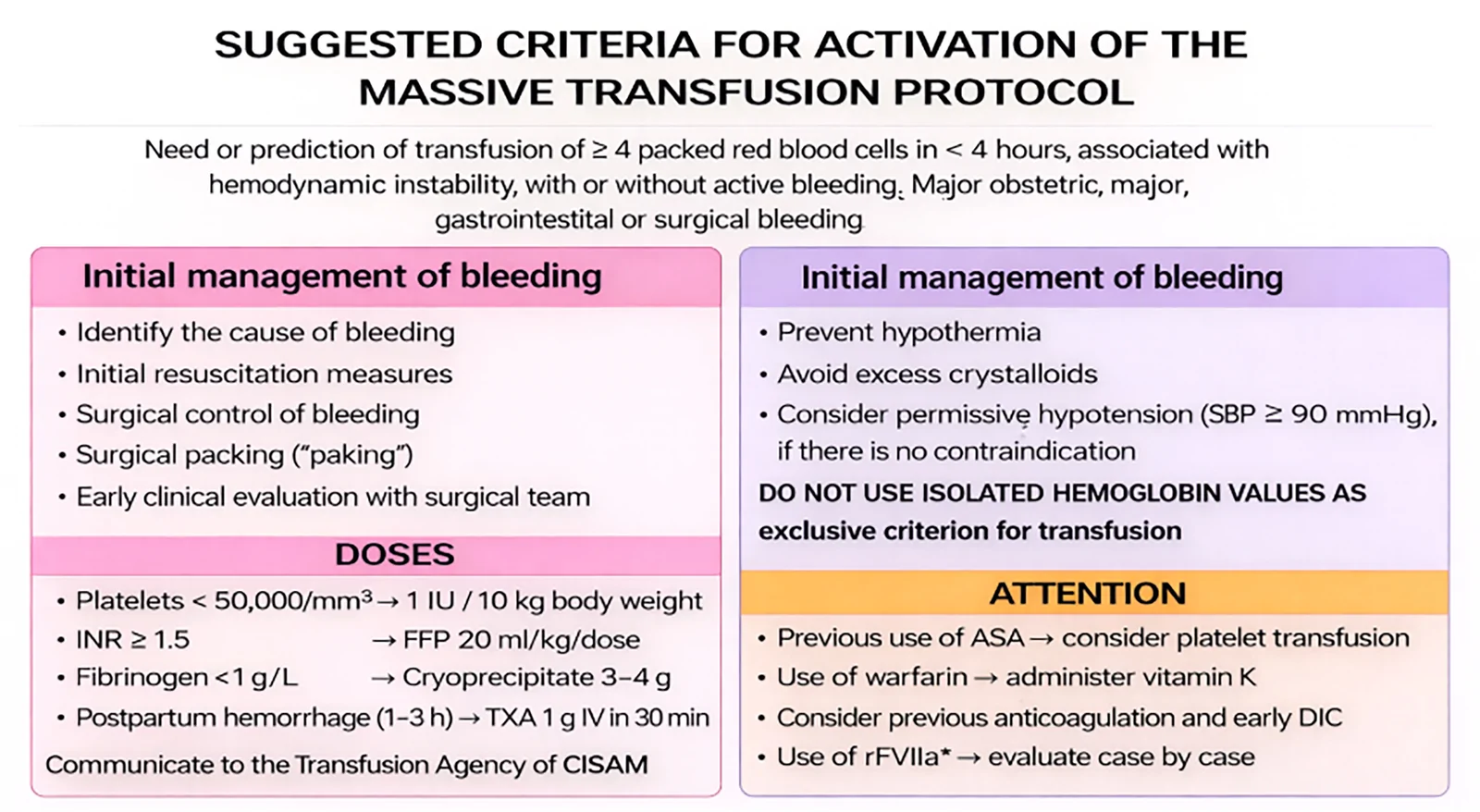
CISAM’s suggested criteria for activation of the massive transfusion protocol 2019.

#### Procedure 3 ‒ Educational measures

Educational measures were implemented through an instant messaging application in addition to presentations of the program in regular clinical meetings at the hospital. CISAM's hemotherapy routines were disseminated through written documentation made available and accessible on an institutional platform (CISAM Wiki) for the hospital's clinical staff. Additionally, information was shared via “WhatsApp” and through periodic meetings with medical staff and residents. The training for the medical team was conducted using digital banners prepared publicly and specifically produced by the researcher. Mastery of the educational material was measured using an icon corresponding to the reading. This project was presented in its entirety or in part at several meetings, including those with the TA team, clinical meetings, conferences with specialists, and senior management staff of the hospital and hemotherapy center. It was also presented at the Symposium of the CH/UPE, in the discipline of hematology and at the rectory.^23^

### Primary outcomes

We analyzed the increase in hemoglobin levels by at least 1 g.dL^-1^ following treatment for iron-deficiency anemia, from the initial consultation to the 30-day follow-up and, when necessary, to the 90-day follow-up.

### Secondary and/or exploratory outcomes

The following outcomes were assessed: 1) The number of Packed Red Blood Cell (PRBC) transfusions received by patients evaluated at the PBM outpatient clinic who underwent hysterectomy or myomectomy during the study period; 2) The number of PRBC transfusions before and after implementation of the PBM program; 3) The total number of activations of the hemorrhage management protocol and the number of blood components administered; and 4) The icon indicating the participants reading of the educational materials distributed through electronic channels.

### Statistical analysis

PBM outpatient clinic: Considering that the service conducts approximately 250 gynecological surgeries monthly, and that 30% of these patients will present with anemia, according to the literature, we included all women that met criteria of AUB and anemia from myomas in the preoperative period of fibroid repair. A convenience sample of 36 patients was used for this study.^24^ Data were collected during consultations at the PBM outpatient clinic using an Excel spreadsheet. The data were periodically inputed and later analyzed using STATA 14.^25^ The evaluation conducted among the treated patients was unmatched, as a PBM outpatient clinic did not exist before 2019. We examined each patient included in the study on a case-by-case basis and described the results. Categorical variables are expressed as absolute and relative frequencies, whereas quantitative variables are expressed as means and standard deviations.^25^

Patients with gynecological conditions scheduled for elective surgery for the treatment of uterine fibroids were admitted to the gynecology ward, underwent surgery in the operating room, and were subsequently transferred to the post-anesthesia care unit and then to the gynecology ward until discharge. After mapping this patient pathway, we analyzed the total number of PRBC transfusions administered to adults throughout the hospital and stratified the data by an adult-care unit. A total of 4,688 PRBC transfusions were recorded during the study period, of which 1,989 were administered to adult patients between January 1, 2016, and December 31, 2023. We also compared the number of transfusions by the hospital unit before and after the implementation of the PBM program, using December 31, 2019, as the cutoff date.

To assess the temporal trend in the mean annual number of PRBC transfusions among adult patients at CISAM from 2016 to 2023, with December 31, 2019, as the intervention milestone, we performed a simple linear regression model using calendar year as the explanatory variable. Given the limited number of annual observations, we conducted an exploratory segmented trend analysis, dividing the time series into two periods (2016–2019 and 2020–2023) and estimating the regression coefficient for each period.^25^

### Implementation of the hemorrhage management protocol

We described numbers of MPT activations, PRBC, and FFP used and presented them as media and standard deviation.

Anonymized data are deposited in Mendeley Dataset with doi: 10.17632/4whnt9x9dh.2 and doi: 10.17632/hd6kg52v9t.2, which has laboratory data.

### Ethical considerations

This project was approved by the Research Ethics Committee of CISAM (CAAE number: 24402719.00000.5191) in 2019. Each patient’s identity was preserved. All the patients signed an informed consent form.

## Results

### Procedure 1 – PBM outpatient clinic

Overall, 57 patients attended the outpatient clinic, after approval of this study by the ethical committee. Of these, 40 were eligible and provided written informed consent; however, four patients were excluded after consent was obtained ‒ two because they were not anemic at presentation and two due to the absence of a diagnosis of uterine myomatosis. Consequently, 36 patients were included in the study (Fig. 3). From those 36 patients, 35 had clinical characteristics documented; 33 had previously been treated with iron or transfusions or both, as described in Table 1. Hemoglobin (Hb) values were available for 31 patients at the first appointment to PBM Outpatient Clinics. Twenty – two returned after 30 days to the second appointment and just 7 needed a third appointment, 90 days after.

**Figure 3:**
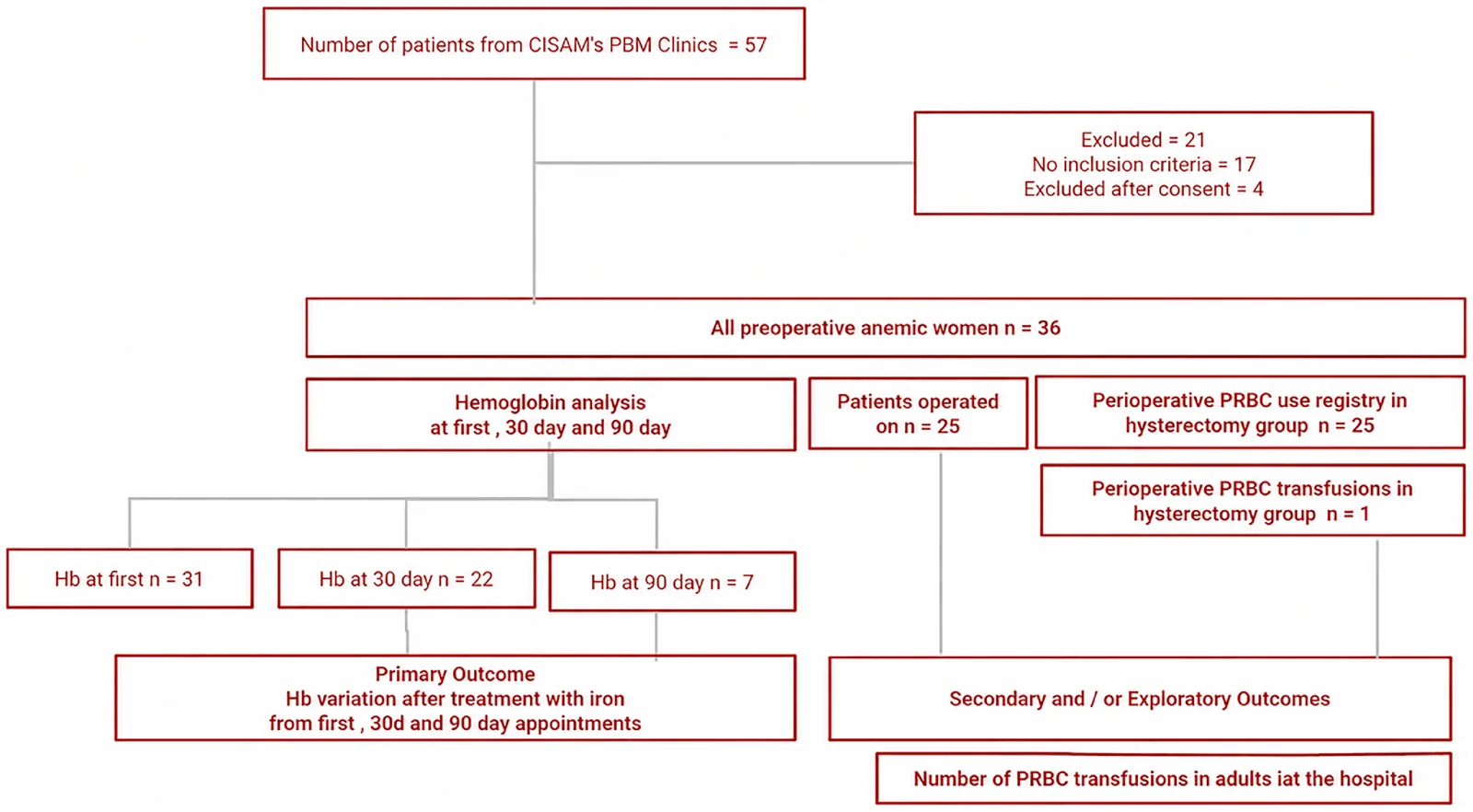
Flowchart of patients from PBM Clinics of Centro Universitário Integrado de Saúde Amaury de Medeiros.

**Table 1:** Clinical characteristics of patients included in the study at the patient blood management outpatient clinic of the Amaury de Medeiros Integrated University Health Center from January 2019 to 2022^a^.

Features	Number (%)
Number of respondents	36 patients^c^
Age (years ‒ Mean ± SD)	42.9 ± 6.8
Conducted surgery	25 (69.4%)
Median time between 1^st^ consultation and surgery (days)^b^	90
Postoperative bleeding	1 (4.0%)
Clinically indicated PRBC transfusion within 48h before surgery^d^	1 (4.0%)
Clinical characteristics (35 patients)	
Irregular cycles	22 (62.9%)
Proiomenorreia	8 (22.9%)
Days per cycle > 7 days	22 (66.7%)
Use of night pad	31 (91.2%)
Duration of anemia, months (3 patients)^b^	24 (11–90)
Previous anemia	22 (66.7%)
Family member with anemia member	4 (11.8%)
Malaise	21 (61.8%)
Daytime cold	12 (36.4%)
Reduced labor	18 (52.9%)
Pica	11 (33.3%)
Does not climb stairs	20 (60.6%)
Restless leg syndrome	9 (27.3%)
Allergies	2 (6.1%)
Weight loss	7 (20.6%)
Shudder	3 (8.8%)
Pyrosis	6 (17,6%)
Bowel habit	1 (2.9%)
Type of previous treatment (33 patients)^e^	
Transfusion	11 (33.3%)
Oral iron	19 (57.6%)
Intravenous iron	12 (36.4%)
Referred for intravenous iron therapy^f^	11 (30.6%)

^# #^17 were referred by an anesthesiologist (48.6%) and 18 by a gynecologist (51.4%).

^# # #^None of the patients who underwent surgery had transfusions until discharge, and no patient reported fever or pruritus.

a Outpatient clinic was conducted by another hematologist during the year of 2023, who did not included patients in this study.

b Median (P25–P75).

c Clinical characteristics of 35 patients ‒ One of the 36 patients had no clinical data but had treatment and surgery information.

d This transfusion was administered for symptomatic anemia and was not intended as prophylactic preoperative transfusion to facilitate surgery.

e Type (s) of treatment(s) before the first appointment in outpatients’ anemia clinic was available for 33 patients.

f Among the patients who were referred for iron therapy, two underwent two sessions, four underwent six sessions, and three underwent 10 sessions; two patients had unavailable data.

Primary outcome – There was a mean increase in Hb levels of 2.48 (95% Confidence Interval: 1.8–3.16, p < 0.001) between the first (Hb1) and second (Hb2) visits to the PBM outpatient clinic for the 22 patients who returned to the 30-day appointment. And a mean increase in Hb levels of 2.53 (95% Confidence Interval:1.39–3.67, p = 0.002) between the first (Hb1) and third (Hb3) for 7 patients who needed an appointment on the 90^th^ day (Table 2).

**Table 2:** Descriptive measures of hemoglobin levels available for the first, 30-day and 90-day appointments of patients seen at the PBM outpatient clinic of CISAM/UPE between 2019 and 2023.

Hemoglobin levels	Mean ± SD
First consultation – 31 patients^a^	8.4 ± 1.5
Second visit (30 days) – 22 patients^b^	10.7 ± 1.3
Third visit (90 days) – 7 patients^c^	11.3 ± 1.3
Comparison of 1^st^ and 2^nd^ visit (22 patients)	
p-value	< 0.001
Mean increase (95% CI)	2.48 (1.8 to 3.16)
Patients with an increase of ≥ 1 g.dL^-1^, n (%)	19 (86.4%)
Comparison of 1^st^ and 3^rd^ visit (7 patients)	
p-value	0.002
Mean increase (95% CI)	2.53 (1.39 to 3.67)
Patients with an increase of ≥ 1 g.dL^-1^, n (%)	7 (100%)

CI, Confidence Interval; PBM, Patient Blood Management; CISAM, Centro Universitário Integrado de Saúde Amaury de Medeiros; UPE, Universidade de Pernambuco.

a Five patients had on hemoglobina value avaiable for ther first appointment.

b Twenty – two patients returned to 30-day appointment.

c Seven patients returned to the 90-day appointment.

Secondary outcome: Overall, 69.4% of the treated patients underwent the surgical procedure at CISAM. From that 36% who weren’t operated on, some stayed waiting for surgery and other lost follow up. Only one patient experienced postoperative bleeding (4%), and one patient required a PRBC transfusion (4%) due to symptomatic anemia. Therefore, the PRBC transfusion was indicated based on the patient’s symptoms and was not intended to facilitate or enable surgical intervention. No other transfusions were required before, during, or after the surgery (Fig. 4).

**Figure 4:**
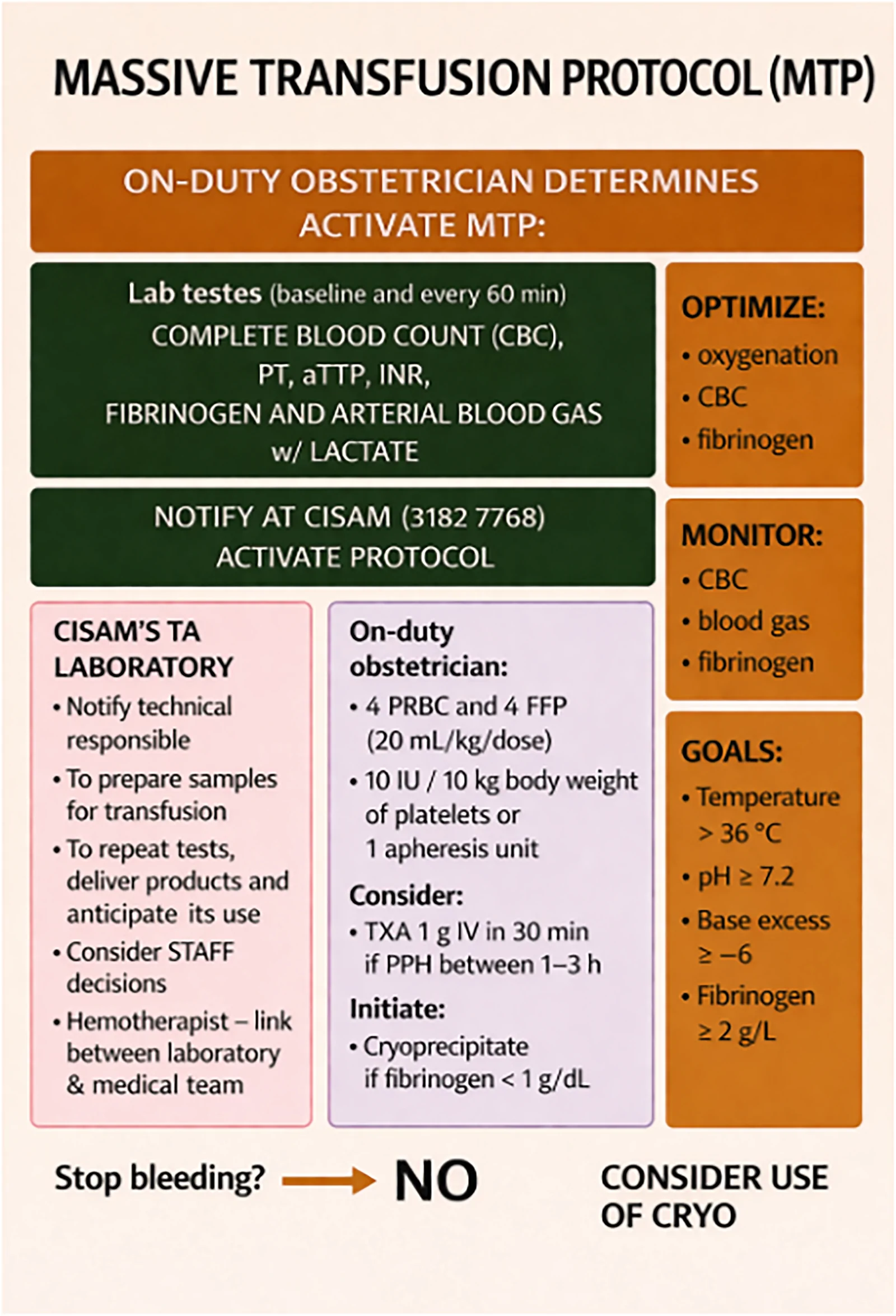
CISAM’s massive transfusion protocol flow chart 2019.

### Analysis of the number of PRBC transfusions in adults

There was a numerical decrease in the number of PRBC transfusions in adults at CISAM. It was 257 (221–375) before January 2020 and 199 (193–204) after that. This trend had a mean reduction of -77.4 (p = 0.253) before January 2020 and maintained its behavior from 2020 to 2023 (1.9; p = 0.686). An expressive reduction occurred at rooming-in unit (-17.8; p = 0.005) and in delivery room (-46.8; p = 0.088). In the emergency room, where we have more severely bleeding patients, we observed higher numbers (p = 0.094) (Table 3; Supplementary Fig. S1).

**Table 3:** Regress coefficient for trend of number of PRBC transfusions in adults during two periods at the Amaury de Medeiros Integrated University Health Center from 2016 to 2019 and 2020 to 2023.

Setting (s)	From 2016 to 2019	From 2020 to 2023
	β (p-value)	β (p-value)
CISAM	−77.4 (0.253)	1.9 (0.686)
Gynecology clinic	−19.2 (0.391)	−0.7 (0.749)
Delivery Room	−46.8 (0.088)	4.0 (0.404)
**Emergency Room**^a^	21 (0.094)	1.8 (0.782)
Surgery Room	−9.4 (0.446)	1.8 (0.211)
**Post-anesthetic care unit**	−6.9 (0.512)	−2.5 (0.305)
**High risk patients’ room**^b^	2.0 (0.555)	−0.4 (0.742)
**Rooming-in**^c^	**-17.8 (0.005)**	−1.2 (0.106)
**Newborn skin-to-skin contact with mother**^c^	−0.3 (0.845)	−0.9 (0.712)

CISAM, Centro Universitário Integrado Amaury de Medeiros.

**** Newborn skin-to-skin contact is a care practice in which the newborn is placed in direct skin contact with the mother.

a Emergency Room is called or Triage at CISAM.

b High risk patients’ room is the yard for high-risk inpatient pregnant women.

c Rooming-in is the yard where mother and babies stay together.

### Procedure 2 ‒ Implementation of the hemorrhagic management protocol

Overall, 4688 transfusions were performed at CISAM (adults and pediatrics). Among these, 197 FFP units were transfused in adults, with the transfusion of 89 units occurring before the implementation of the hemorrhage care protocol and 65 occurring after this date. The mean number of FFP unit transfusions was 22.5 (20–29.5) before the protocol was implemented and 23 (17.5–31.5) after (p = 1.00) (Supplementary Fig. S2). To sensitize retrospective search of data for cases of Massive Transfusion Protocol (MTP) activation, we started searching data from all records of transfused patients with ≥ 3 PRBC units or any amount of FFP between 2019 and December 2023 (Supplementary Table S1). This initial search resulted in 69 patients who have received ≥ 3 PRBC units or any amount of FFP. Six of them (6/69) received 4 or more PRBC units and, at least a FFP unit infusion, which met activation criteria according to MTP designed for CISAM at that time, and 5 (5/6) of MTP were to treat PPH (Supplementary Tables S2‒S3). Activation of MTP for PPH (5 cases) used a total of 23 PRBC and 11 FFP units (mean of 4.6 PRBC units and 2.2 FFP units per patient). The ratio of PRBC to FFP units was 2.09:1. There was one transfusion of platelets concentrate, and no records of cryoprecipitate utilization in any of the situations. There was an increase in thawed and unused FFP units from 6 units by 2019 to 11 units by 2020. Tranexamic acid is used in all cases of PPH at CISAM. There was no registry of death among those five patients.

### Procedure 3 – Educational measures

A total of 546 healthcare professionals received and read messages sent through WhatsApp to service groups, including managers, medical managers, working groups of PBM at CISAM and CH/UPE, and resident doctors. PBM and CISAM protocols were presented in 24 meetings with around 1548 people in the audience.

## Discussion

We observed a significant change in care practices in this study. Reducing the number of PRBC transfusions improves clinical practice. The educational strategy using available and low-cost resources, such as “WhatsApp”, was important for continuous education of health professionals and in the training of the team local multidisciplinary approach of the PBM. The HMP turned MPT policy into a systematic one and modified transfusional routines of staff during PPH. We used the available oral and intravenous iron compounds, resulting in an adequate increase in the Hb levels of patients treated at the PBM outpatient clinic. Preoperative anemia impairs the outcomes of women scheduled for gynecological surgeries, and transfusions do not correct this risk; however, they are associated with 61% and 16% increase in mortality and compound morbidity, respectively.^5^ The impact of anemia on the management of patients hospitalized for leiomyoma was evaluated by Antunes et al., who concluded that the early identification and treatment of anemia improved care. Fajardo et al. (2023) evaluated 26,299 patients with fibroids who underwent myomectomy by laparotomy or video laparoscopy and concluded that an increased hematocrit was associated with a lower risk of blood transfusions. Therefore, optimization of Hb levels using iron replacement without transfusions in the pre-, intra-, and postoperative periods is consistent with the most recent literature and first PBM pillar, as recommended by the WHO.^1^^,^^9^^,^^26^

The implementation of written protocols, establishment of a clinic specialized in the treatment of anemia, and the promotion of frequent continuous education were fundamental for modifying local prescriptive habits, resulting in a reduction in PRBC transfusions which was sustained throughout the subsequent observation period. Education may help to systemically reduce the number of transfusions. These data reflect changes in the local culture linked to the pillars of PBM and its accelerators. These changes were made possible through accessible technologies in our service. Therefore, the implementation of PBM is feasible in scenarios with limited resources, such as ours.^2^^,^^23^ Hemorrhage management protocols are recommended by current health regulations in Brazil. The implementation of an easy-to-understand protocol, disseminated through instant messaging, and widely known by the institution's professionals, is fundamental for improving the care of patients with PPH. We found that multiple transfusions at CISAM were unrelated to PPH and instead they were associated to other diagnosis. The mean transfusion ratio (PRBC:FFP) was 2.09:1 per patient with PPH with just one platelet transfusion, different from international protocols and possibly reflecting resource limitations or protocol maturity.^21^^,^^27^

This study has some limitations. The relatively short study period and limited sample size may have reduced the statistical power of the study, potentially limiting the ability to detect statistically significant differences between the study periods. However, the absence of transfusions in the elective preoperative setting, overall reduction in PRBC transfusions, and their maintenance for all the years after implementation may demonstrate program consistency. The activities of the PBM outpatient clinic were suspended in March 2020 and resumed in August 2021 because of the COVID-19 pandemic, which may be an important temporal confounder. There was no established practice of referring patients to a hematologist preoperatively. In fact, this interruption limited the growth in the number of outpatients evaluated during the study period. Concerning the number of PRBC transfusions, no obvious sustained disruption was observed in the available descriptive data as we reviewed the annual number of surgeries performed at our hospital from 2016 to 2023. The mean number of surgeries was 251 per year from 2016 to 2019 and 266 per year from 2020 to 2023. After excluding 2020, the mean increased to 291 surgeries per year. Therefore, despite the disruption caused by the pandemic period, surgical activity and PRBC transfusion requirements remained stable throughout the study period. It is important to note that this study was not designed to isolate the effect of the pandemic period from the PBM implementation.^28^ Initially, we did not have kits for ferritin measurement, which made it difficult to accurately diagnose iron-deficiency anemia. However, using data from the clinical history of chronic blood loss, we ruled out other diseases. In addition to the complete blood count, it was possible to make a presumptive diagnosis of IDA and treat patients who had increased Hb levels and did not need perioperative transfusions. Despite these drawbacks, we evaluated the ferrokinetics in all patients.^29^ Finally, quantitative blood loss and other perioperative outcomes that could serve as proxy measures of bleeding severity (such as postoperative hemoglobin, hemoglobin decrease, reoperation for bleeding, ICU admission, use of hemostatic agents, or procedure-specific transfusion data) were not routinely collected in our database. Consequently, these outcomes could not be evaluated. Specifically, for the PPH massive transfusion protocol, we analyzed the number of activations and use of blood components.

## Conclusion

Improvements in health care have provided patients with adequate treatment for IDA, which improved results in hemoglobin levels among patients with paired measures. It allowed patients with myomas and with appointments in PBM clinics to undergo surgical procedures without receiving inappropriate prophylactic packed red blood cells PRBC transfusions before hysterectomy, which reduces immediate and late risks associated with the use of blood components. For the institution, PBM outpatient clinic was an innovation because it was the implementation of a new service line, which modified the workflow and the current medical assistance. Additionally, the sustained reduction in the number of PRBC transfusions may support the feasibility of a PBM program in a setting of constraints and may reflect a consistent implementation of the PBM outpatient clinic. The adequate treatment of IDA was possible using resources available in our center. The design of a hemorrhage management protocol was also an advancement. The implementation of these changes, integrating education and clinical practice in a resource-limited setting and leveraging free, widely accessible digital tools, underscores the role of educational activities and it is aligned with the pillars of PBM, which improves care through ethical and evidence-based practices.^2^^,^^30^

In summary, our findings support the feasibility of implementing this model, demonstrate increased hemoglobin levels among patients with paired measurements, show the absence of prophylactic preoperative transfusions in the outpatient PBM cohort undergoing surgery, and describe institutional transfusion trends. Further research is needed to evaluate the cost-effectiveness of such models, particularly given their potential to extend high-impact care to remote regions facing financial and technological constraints.

## Data availability statement

The data that support the findings of this study are available through Correa, Lorena (2026), “Implementing Patient Blood Management in a Brazilian Public Women’s Hospital: a Pre–Post Implementation Study”, Mendeley Data, V2, doi: 10.17632/4whnt9x9dh.2 and through Correa, Lorena (2026), “Implementing Patient Blood Management in a Brazilian Public Women’s Hospital: a Pre–Post Implementation Study - with lab”, Mendeley Data, V2, doi: 10.17632/hd6kg52v9t.2.

## Declaration of generative AI and AI-assisted technologies in the writing process

During the preparation of this work the authors used ChatGPT for translation and for layout of some figures. The text has authors full review, edition and responsibility about it. No AI tool was used for study design, data collection, data analysis, interpretation, or scientific conclusions.

## Institutional research board approval

This project was approved by the Research Ethics Committee of CISAM (CAAE number: 24402719.00000.5191) in 2019 and remains in progress. Each patient’s identity was preserved. All the patients signed an informed consent form.

## Authors’ contributions

Conceptualization, A.N.C, C.C.L; Acquisition and execution, C.C.L, P.A.A.N, A.D.C.A; Analysis, M.U; Interpretation, A.N.C, C.C.L, M.U; Drafting, A.N.C, C.C.L; Revising, all authors.

## Funding

This research did not receive any specific grant from funding agencies in the public, commercial, or not-for-profit sectors.

## Conflicts of interest

The authors declare no conflicts of interest.

## Appendix Supplementary materials

Image, application 1

## Footnotes

Supplementary material associated with this article can be found in the online version at doi:10.1016/j.bjane.2026.844839.
