# Supplementary material for: Implementing patient blood management in a Brazilian public women’s hospital: a pre–post implementation study
Source: Braz J Anesthesiol. 2026 Oct 1;76(6):844839. doi: 10.1016/j.bjane.2026.844839 (PMC13695121; doi:10.1016/j.bjane.2026.844839)
Supplement: Supplementary file 1 [file mmc1.docx]

**BJAN-D-25-00669**

**Supplementary Material**

**Supplementary Table S1** Retrospective analysis of massive transfusion protocols based on the transfusion of ≥ 3 Units of PRBCs and ≥ 1 Unit of FFP.

| **Date** | **Diagnostic** | **Number of** **PRBC Units** | **Number of FFP Units** | **Platelets concentrate transfusion** | **≥ 3 PRBC units** | **≥ 1 FFP^b^ units** | **PPH** |
| --- | --- | --- | --- | --- | --- | --- | --- |
| Mar 20, 2019 | Placental abruption | 3 | 1 | 0 | Yes | Yes | Yes |
| Jun 10, 2019 | Curettage (retained products of conception) | 4 | 1 | 0 | Yes | Yes | Yes |
| Dec 13, 2019 | Postpartum hemorrhage | 6 | 2 | 0 | Yes | Yes | Yes |
| Jun 4, 2020 | Ruptured ectopic pregnancy | 3 | 1 | 0 | Yes | Yes | No |
| Jul 24, 2020 | Postpartum hemorrhage | 3 | 3 | 0 | Yes | Yes | No |
| Aug 16, 2020 | Severe anemia | 3 | 2 | 0 | Yes | Yes | No |
| Jan 17, 2021 | Hemorrhagic shock after cesarean section | 3 | 2 | 1 | Yes | Yes | No |
| Feb 23, 2021 | Hemorrhagic shock after cesarean section | 5 | 4 | 1 | Yes | Yes | Yes |
| Jun 18, 2021 | No diagnosis | 3 | 1 | 0 | Yes | Yes | No |
| Apr 26, 2022 | Hemorrhagic shock after cesarean section | 4 | 3 | 0 | Yes | Yes | Yes |
| May 21, 2022 | Ectopic pregnancy and anemia | 3 | 2 | 0 | Yes | Yes | No |
| Nov 1, 2022 | Neoplasm of vagina | 3 | 3 | 0 | Yes | Yes | No |
| Jan 1, 2023 | Hemorrhagic shock after cesarean section (accreta placenta) | 4 | 1 | 0 | Yes | Yes | Yes |
| Feb 1, 2023 | Hemorrhagic shock after abdominal hemorrhage | 4 | 2 | 0 | Yes | Yes | No |

PRBC, Packed Red Blood Cell; FFP, Fresh frozen plasma; PPH, Postapartum Hemorrhage.

**Supplementary Table S2** Use of blood components in the massive transfusion protocol for postpartum hemorrhage at Centro Integrado de Saúde Amaury de Medeiros, 2019–2023.

| **Date** | **Diagnostic** | **PRBC units** | **FFP units** | **Platelets concentrate transfusion** |
| --- | --- | --- | --- | --- |
| Jun 10, 2019 | Curettage (retained products of conception) | 4 | 1 |  |
| Dec 13, 2019 | Postpartum hemorrhage | **6** | 2 |  |
| Feb 23, 2021 | Hemorrhagic shock after cesarean section | **5** | 4 | 1 |
| Apr 26, 2022 | Hemorrhagic shock after cesarean section | 4 | 3 | 0 |
| Jan 1, 2023 | Hemorrhagic shock after cesarean section (accreta placentae) | 4 | 1 | 0 |

PRBC, Packed Red Blood Cell; FFP, Fresh frozen plasma.

**Supplementary Table S3** Massive Transfusion Protocol Activations for Postpartum Hemorrhage from 2019 to 2023 at Centro Integrado de Saúde Amaury de Medeiros.

| **Year** | **Activations** |
| --- | --- |
| 2019 | 2 |
| 2020 | 0 |
| 2021 | 1 |
| 2022 | 1 |
| 2023 | 1 |

**Supplementary Figure S1** Monitoring of the number of packed red blood cells transfusions in adults at the Amaury de Medeiros Integrated University Health Center from 2016 to 2023. *Mean reduction per year of 77 transfusions (p = 0.253) observed from 2016 to 2019. **Constant number of transfusions (p = 0.686) observed from 2020 to 2023.

**Supplementary Figure S2** Number of Fresh Frozen Plasma (FFP) transfusions in adults at the Amaury de Medeiros Integrated University Health Center from 2016 to 2023.
